# Risk of Suicide After Cancer Diagnosis in England

**DOI:** 10.1001/jamapsychiatry.2018.3181

**Published:** 2018-11-21

**Authors:** Katherine E. Henson, Rachael Brock, James Charnock, Bethany Wickramasinghe, Olivia Will, Alexandra Pitman

**Affiliations:** 1 National Cancer Registration and Analysis Service, Public Health England, Wellington House, London, United Kingdom; 2 Bury St Edmunds GP Specialty Training Programme, West Suffolk Hospital, Bury St Edmunds, United Kingdom; 3 Transforming Cancer Services Team for London, Skipton House, London, United Kingdom; 4 Department of Surgery, West Suffolk National Health Service Foundation, Bury St Edmunds, Suffolk, United Kingdom; 5 UCL Division of Psychiatry, University College London, London, United Kingdom; 6 Camden and Islington National Health Service Foundation Trust, St Pancras Hospital, London, United Kingdom; 7 St George’s University Hospitals National Health Service Foundation Trust, London, United Kingdom

## Abstract

**Importance:**

A diagnosis of cancer carries a substantial risk of psychological distress. There has not yet been a national population-based study in England of the risk of suicide after cancer diagnosis.

**Objectives:**

To quantify suicide risk in patients with cancers in England and identify risk factors that may assist in needs-based psychological assessment.

**Design, Setting, and Participants:**

Population-based study using data from the National Cancer Registration and Analysis Service in England linked to death certification data of 4 722 099 individuals (22 million person-years at risk). Patients (aged 18-99 years) with cancer diagnosed from January 1, 1995, to December 31, 2015, with follow-up until August 31, 2017, were included.

**Exposures:**

Diagnosis of malignant tumors, excluding nonmelanoma skin cancer.

**Main Outcomes and Measures:**

All deaths in patients that received a verdict of suicide or an open verdict at the inquest. Standardized mortality ratios (SMRs) and absolute excess risks (AERs) were calculated.

**Results:**

Of the 4 722 099 patients with cancer, 50.3% were men and 49.7% were women. A total of 3 509 392 patients in the cohort (74.3%) were aged 60 years or older when the diagnosis was made. A total of 2491 patients (1719 men and 772 women) with cancer died by suicide, representing 0.08% of all deaths during the follow-up period. The overall SMR for suicide was 1.20 (95% CI, 1.16-1.25) and the AER per 10 000 person-years was 0.19 (95% CI, 0.15-0.23). The risk was highest among patients with mesothelioma, with a 4.51-fold risk corresponding to 4.20 extra deaths per 10 000 person-years. This risk was followed by pancreatic (3.89-fold), esophageal (2.65-fold), lung (2.57-fold), and stomach (2.20-fold) cancer. Suicide risk was highest in the first 6 months following cancer diagnosis (SMR, 2.74; 95% CI, 2.52-2.98).

**Conclusions and Relevance:**

Despite low absolute numbers, the elevated risk of suicide in patients with certain cancers is a concern, representing potentially preventable deaths. The increased risk in the first 6 months after diagnosis may indicate an unmet need for psychological support. The findings of this study suggest a need for improved psychological support for all patients with cancer, and attention to modifiable risk factors, such as pain, particularly in specific cancer groups.

## Key Points

**Question:**

What is the risk of suicide after cancer diagnosis in England, and which subgroups of patients are most at risk?

**Findings:**

In this population-based study of 4 722 099 adult patients with cancer, a 20% increased risk of suicide compared with the general population was noted, corresponding to 0.19 excess deaths per 10 000 person-years. Patients with mesothelioma, pancreatic, esophageal, and lung cancer had the highest risk.

**Meaning:**

The first 6 months after diagnosis of cancer has been shown to be a critical period, identifying the time during which cancer care pathways should pay particular attention to the psychological health needs of the patients; as suicide is hard to predict, all patients should receive improved psychological support.

## Teaser

This population-based study examines the risk of suicide in patients in England after diagnosis of cancer, including cancer type and stage, time since diagnosis, and sociodemographic factors.

## Introduction

A diagnosis of cancer can cause substantial psychological distress.^1^ Patients may fear death; pain; adverse effects of treatment, such as disfigurement or loss of function; or alterations in their family and community roles.^1^ This distress may have a role in the development of suicide ideation: previous evidence from systematic reviews has shown an increased risk of suicide among patients with cancer.^2,3^

In the United Kingdom, the prevalence of patients with a cancer diagnosis is growing, driven by increased survival, improved cancer treatments, and expanding cancer screening programs.^4^ Despite political and financial prioritization of diagnostic and therapeutic cancer services and public health campaigns aimed at facilitating earlier diagnosis of cancer, there are substantial historical resource constraints for mental health services. In addition, acute care hospitals and mental health services remain disconnected.^5,6^

Although the absolute risk of suicide may be low compared with the risks for other causes of death in patients with cancer, these deaths are potentially preventable. They are also a proxy marker of substantial psychological distress in patients with cancer. In 2015, the Cancer Taskforce report highlighted that better management of depression could improve patient outcomes, affecting quality of life as well as adherence to treatment, thereby having an influence on survival.^7^ To target psychological screening and support for the patients in greatest psychological distress and at risk of suicide, we need to understand variation in risk of suicide by patient group as defined by, for example, age, sex, and cancer type.

To our knowledge, this study is the first national population-level analysis of suicide among patients with cancer in England. Using longitudinal data from the National Cancer Registration and Analysis Service, linked to Office for National Statistics death certification data, we examined the risk of suicide in patients with a cancer diagnosis compared with that of the general population. We further analyzed the risk in terms of cancer type and stage, time since diagnosis, and sociodemographic factors.

## Methods

### Study Population

Patients diagnosed with a malignant tumor (*International Statistical Classification of Diseases*, *10th Revision [ICD-10]* codes C00-C97, excluding C44, nonmelanoma skin cancer, using an internal mapping system for all *ICD* revisions) during January 1, 1995, to December 31, 2015, and aged 18 to 99 years at diagnosis, were identified from Public Health England’s national cancer registration database. All patients were residents of England at the time of their diagnosis. Patients identified as having cancer on their death certificate alone were excluded as these patients were not aware of their diagnosis. Individuals diagnosed on the same day as their death contributed 1 day to the risk period. For individuals with more than 1 cancer diagnosis, the date and diagnosis of their most recent cancer was selected using the groupings available in eTable 1 in the Supplement. The study was exempt from gaining individual consent to participate having obtained approval from the UK Patient Information Advisory Group (now the Confidentiality Advisory Group, under Section 251 of the National Health Service Act 2006 [PIAG 03(a)/2001]). Ethical approval was not applicable.

Cause of death was ascertained through tracing by the Office for National Statistics. All deaths in patients with cancer that received a verdict of suicide or an open verdict at the inquest were selected. An open verdict is a legal decision that records a death, but does not state the cause. These were identified using *ICD-10* codes for suicide (X60-X84 and Y87.0) and open verdicts (Y10-Y34 [excluding Y33.9, which is now called U309] and Y87.2 [event of undetermined intent]) and their *International Classification of Diseases, Ninth Revision (ICD-9) *equivalents: E950-E959 for suicide and self-inflicted injury and E980-E989 (excluding E988.8) for undetermined death. Deaths were selected using the final underlying cause of death, as per the World Health Organization guidelines.^8^

The income deprivation quintile was derived by linking each patient’s postcode at diagnosis (at the Lower Super Output Area geography) to the income domain of the Index of Multiple Deprivation.^9^ Equal population quintiles were derived across England from the income domain score, and each patient with cancer was assigned the income deprivation quintile of their local geography. Ethnicity was self-reported, recorded in hospital patient administration systems, and submitted to the cancer registry. If a patient’s ethnicity was unknown, it was supplemented by information on self-reported ethnicity as recorded in the Hospital Episode Statistics data set. As ethnicity may be recorded differently during each hospital admission, the modal value was used in cases of discrepancy, preferring the most recent ethnicity documented in the event of a tied modal value.

The cancer registry operates on a benchmark of 70% completeness for stage. Completeness of stage has been deemed sufficient for analysis since 2012^10^; thus, stage at cancer diagnosis was selected for a subgroup analysis.

### Statistical Analysis

Each patient was entered into the analysis on their date of cancer diagnosis and was censored at the date of death, date when lost to follow-up, or August 31, 2017. Standardized mortality ratios (SMRs) and absolute excess risks (AERs) were calculated using standard cohort techniques.^11^ The SMR was defined as the number of deaths by suicide observed in the cancer population / number of expected deaths by suicide. The AER was defined as the ([number observed − number expected] / [person-years at risk]) × 10 000. These measures estimate the proportional increase in the suicide rate compared with rates in the general population and the absolute excess suicide rate compared with the general population, respectively. Population-based expected deaths were derived from age (5-year groups), sex, and calendar-year (1-year groups) specific death rates for England.^12^

Tests for heterogeneity were performed using likelihood ratio tests based on Poisson regression models. This test compared the deviance of a model including the factor of interest to the deviance of a model without the factor of interest. Statistical significance was defined as 2-sided with significance level set at *P* < .05.

Multivariable Poisson regression models were used to evaluate the simultaneous effect of the factors of interest. All factors were fitted in the regression model with no interaction terms. These factors were sex, cancer type, deprivation, race/ethnicity, age at cancer diagnosis, year of diagnosis, attained age, and follow-up period. Attained age and follow-up period were never fitted in the same model due to strong collinearity. These multivariable models produced relative risks (with an offset of the log of the expected number of deaths), which were the ratio of SMRs adjusted for all factors, and relative excess risks (with an offset of the log of the person-years and a link function^13^), which were the ratio of AERs adjusted for all factors.^11^ The heterogeneity tests from the multivariable models are presented herein, and the estimates are presented in eTable 2 in the Supplement. All calculations were performed using Stata, version 13 (StataCorp).

## Results

### Patient Characteristics and Overall Findings

A total of 4 722 099 individuals (50.3% men and 49.7% women) aged 18 to 99 years received a diagnosis of cancer during the study period. A total of 3 509 392 patients in the cohort (74.3%) were aged 60 years or older at the time of diagnosis. A total of 2491 patients (1719 men and 772 women) with cancer were recorded to have died by suicide over a follow-up period up to 22 years. This number represented 0.08% of all deaths. The cohort characteristics are summarized in Table 1. The cohort contributed a total of 22 036 669 person-years, and the mean (SD) and median follow-up were 4.67 (5.33) and 2.52 years, respectively. The SMR for all cancers combined was 1.20 (95% CI, 1.16-1.25) and the AER per 10 000 person-years at risk was 0.19 (95% CI, 0.15-0.23) (Table 2). An elevated risk was demonstrated in each deprivation quintile, where no significant heterogeneity was observed (Table 3). There were variations in the association between suicide risk and attained age, with significant heterogeneity overall (Table 3 and eTable 3 in the Supplement).

**Table 1.** Characteristics of Study Cohort

Characteristic	Patients With Cancer, No.	Deaths			
		Suicide, No.	Total, No.	% Suicide of Total	% Suicide of Category
Total	4 722 099	2491	3 078 843	0.08	
Sex					
Male	2 376 908	1719	1 652 298	0.10	69.01
Female	2 345 191	772	1 426 545	0.05	30.99
Time from cancer diagnosis					
0-5 mo	1 253 682	540	1 238 800	0.04	21.68
6-11 mo	398 010	241	396 936	0.06	9.67
12-23 mo	487 700	329	430 204	0.08	13.21
24-35 mo	394 513	261	234 198	0.11	10.48
3-4 y	556 897	316	274 653	0.12	12.69
5-9 y	846 352	521	323 021	0.16	20.92
≥10 y	784 945	283	181 031	0.16	11.36
Age at death (attained age), y					
18-29	19 271	15	8260	0.18	0.60
30-49	265 245	267	114 831	0.23	10.72
50-59	493 004	422	259 911	0.16	16.94
60-69	956 714	585	578 161	0.10	23.48
70-79	1 451 587	647	954 159	0.07	25.97
≥80	1 536 278	555	1 163 521	0.05	22.28
Last primary cancer					
Bladder	172 359	120	127 291	0.09	4.82
Breast	744 817	339	293 606	0.12	13.61
Cancer of unknown primary	177 747	29	170 004	0.02	1.16
CNS (including brain)	70 906	28	62 276	0.04	1.12
Cervix	50 696	23	20 258	0.11	0.92
Colorectal	578 270	349	392 959	0.09	14.01
Head and neck	167 307	176	89 713	0.20	7.07
Hodgkin lymphoma	25 155	25	7307	0.34	1.00
Kidney and unspecified urinary organs	122 974	72	78 693	0.09	2.89
Leukemia	116 707	53	82 239	0.06	2.13
Liver	51 800	10	47 900	0.02	0.40
Lung	613 772	184	577 911	0.03	7.39
Melanoma	161 734	97	49 808	0.19	3.89
Mesothelioma	36 062	20	35 059	0.06	0.80
Multiple myeloma	73 191	41	55 836	0.07	1.65
Non-Hodgkin lymphoma	170 764	113	101 264	0.11	4.54
Esophagus	122 132	57	111 553	0.05	2.29
Other malignant neoplasms	133 186	78	93 364	0.08	3.13
Ovary	112 384	33	76 763	0.04	1.32
Pancreas	121 207	33	117 298	0.03	1.32
Prostate	594 521	444	302 584	0.15	17.82
Sarcoma	28 712	14	15 951	0.09	0.56
Stomach	128 965	59	117 763	0.05	2.37
Testis	34 630	58	2835	2.05	2.33
Uterus	112 101	36	48 608	0.07	1.45
Deprivation					
1, Least	922 123	505	533 157	0.09	20.27
2	999 928	558	620 458	0.09	22.40
3	984 525	518	645 294	0.08	20.79
4	940 242	492	648 231	0.08	19.75
5, Most	875 281	418	631 703	0.07	16.78
Race/ethnicity					
White	3 087 922	1521	1 913 960	0.08	61.06
Mixed	7654	5	3090	0.16	0.20
Asian	55 955	16	26 007	0.06	0.64
Black	46 039	11	22 213	0.05	0.44
Other	35 148	19	18 247	0.10	0.76
Not stated	703 527	435	535 181	0.08	17.46
Unknown	785 854	484	560 145	0.09	19.43
Age at cancer diagnosis, y					
18-29	63 059	49	12 048	0.41	1.97
30-49	465 201	368	151 316	0.24	14.77
50-59	684 447	492	318 169	0.15	19.75
60-69	1 167 097	630	676 827	0.09	25.29
70-79	1 354 726	624	1 032 533	0.06	25.05
≥80	987 569	328	887 950	0.04	13.17
Year of cancer diagnosis					
1995-1999	995 756	486	836 543	0.06	19.51
2000-2004	1 056 151	736	795 971	0.09	29.55
2005-2009	1 149 793	681	734 519	0.09	27.34
2010-2015	1 520 399	588	711 810	0.08	23.60

Abbreviation: CNS, central nervous system.

**Table 2.** Suicide SMRs and AERs per 10 000 Person-Years at Risk According to Last Primary Cancer

Last Primary Cancer	Observed/Expected	SMR (95% CI)	AER per 10 000 (95% CI)
Total	2491/2072	1.20 (1.16 to 1.25)	0.19 (0.15 to 0.23)
**Significantly Increased Suicide Rate Among Patients With Cancers vs General Population**			
Mesothelioma	20/4	4.51 (2.91 to 7.00)	4.20 (1.84 to 6.57)
Pancreas	33/8	3.89 (2.77 to 5.48)	2.89 (1.56 to 4.22)
Esophagus	57/22	2.65 (2.04 to 3.43)	1.83 (1.07 to 2.60)
Lung	184/72	2.57 (2.23 to 2.97)	1.56 (1.19 to 1.93)
Stomach	59/27	2.20 (1.71 to 2.84)	1.35 (0.72 to 1.98)
Cancer of unknown primary	29/15	1.98 (1.38 to 2.85)	1.00 (0.26 to 1.74)
Head and neck	176/105	1.67 (1.44 to 1.94)	0.74 (0.46 to 1.01)
CNS (including brain)	28/17	1.61 (1.11 to 2.33)	0.74 (0.01 to 1.48)
Multiple myeloma	41/26	1.57 (1.15 to 2.13)	0.58 (0.09 to 1.08)
Other malignant neoplasms	78/54	1.46 (1.17 to 1.82)	0.44 (0.13 to 0.76)
Colorectal	349/274	1.28 (1.15 to 1.42)	0.28 (0.14 to 0.41)
**Significantly Increased Proportional SMR Among Patients With Cancers vs General Population**			
Non-Hodgkin lymphoma	113/93	1.22 (1.01 to 1.47)	0.23 (−0.01 to 0.46)
**Significantly Decreased Suicide Rate Among Patients With Cancers vs General Population**			
Prostate	444/494	0.90 (0.82 to 0.99)	−0.14 (−0.25 to −0.02)
Melanoma	97/121	0.80 (0.66 to 0.98)	−0.20 (−0.35 to −0.04)
**No Significant Difference in the Suicide Rate Among Patients With Cancers vs General Population**			
Liver	10/6	1.56 (0.84 to 2.90)	0.64 (−0.47 to 1.75)
Ovary	33/27	1.23 (0.87 to 1.73)	0.12 (−0.10 to 0.34)
Kidney and unspecified urinary organs	72/59	1.22 (0.96 to 1.53)	0.24 (−0.07 to 0.55)
Bladder	120/106	1.13 (0.95 to 1.36)	0.16 (−0.08 to 0.41)
Breast	339/313	1.08 (0.97 to 1.21)	0.04 (−0.02 to 0.11)
Cervix	23/22	1.05 (0.69 to 1.57)	0.03 (−0.22 to 0.27)
Leukemia	53/52	1.02 (0.78 to 1.34)	0.02 (−0.28 to 0.32)
Hodgkin lymphoma	25/26	0.95 (0.64 to 1.41)	−0.06 (−0.52 to 0.40)
Uterus	36/39	0.92 (0.67 to 1.28)	−0.04 (−0.19 to 0.11)
Testis	58/71	0.81 (0.63 to 1.05)	−0.36 (−0.76 to 0.04)
Sarcoma	14/18	0.77 (0.45 to 1.30)	−0.26 (−0.72 to 0.19)
*P* value for heterogeneity		<.001	<.001
Adjusted (follow-up) *P* value for heterogeneity^a^		<.001	<.001

Abbreviations: AERs, absolute excess risks; CNS, central nervous system; SMRs, standardized mortality ratios.

^a^ Fully adjusted for sex, cancer type, deprivation, race/ethnicity, age at cancer diagnosis, year of diagnosis (5-year groups), and follow-up period.

**Table 3.** Suicide SMRs and AERs per 10 000 Person-Years at Risk According to Key Patient Characteristics for All Cancers Combined^a^

Characteristic	Observed/Expected	SMR (95% CI)	AER per 10 000 (95% CI)
Sex			
Men	1719/1433	1.20 (1.14 to 1.26)	0.29 (0.21 to 0.37)
Women	772/639	1.21 (1.13 to 1.30)	0.11 (0.06 to 0.15)
*P* value for heterogeneity		.88	<.001
Adjusted (follow-up) *P* value for heterogeneity^b^		.52	<.001
Age at death (attained age), y			
18-29	15/22	0.69 (0.42 to 1.15)	−0.30 (−0.64 to 0.05)
30-49	267/268	1.00 (0.88 to 1.12)	0.00 (−0.14 to 0.13)
50-59	422/348	1.21 (1.10 to 1.33)	0.22 (0.10 to 0.34)
60-69	585/447	1.31 (1.21 to 1.42)	0.25 (0.16 to 0.33)
70-79	647/502	1.29 (1.19 to 1.39)	0.24 (0.15 to 0.32)
≥80	555/485	1.15 (1.05 to 1.24)	0.16 (0.06 to 0.27)
*P* value for heterogeneity		<.001	.01
Adjusted (attained age) *P* value for heterogeneity^c^		<.001	<.001
Deprivation			
1, Least	505/464	1.09 (1.00 to 1.19)	0.08 (−0.01 to 0.17)
2	558/466	1.20 (1.10 to 1.30)	0.18 (0.09 to 0.28)
3	518/431	1.20 (1.10 to 1.31)	0.19 (0.09 to 0.29)
4	492/384	1.28 (1.17 to 1.40)	0.27 (0.16 to 0.37)
5, Most	418/327	1.28 (1.16 to 1.41)	0.27 (0.15 to 0.38)
*P* value for heterogeneity		.07	.06
Adjusted (follow-up) *P* value for heterogeneity^b^		.32	.43
Race/ethnicity			
White	1521/1369	1.11 (1.06 to 1.17)	0.10 (0.05 to 0.16)
Mixed	5/4	1.38 (0.57 to 3.31)^d^	0.37 (−0.81 to 1.55)^d^
Asian	16/26	0.62 (0.38 to 1.01)	−0.36 (−0.65 to −0.07)
Black	11/24	0.45 (0.25 to 0.81)	−0.57 (−0.85 to −0.29)
Other	19/16	1.16 (0.74 to 1.82)	0.15 (−0.34 to 0.64)
Not stated	435/309	1.41 (1.28 to 1.55)	0.39 (0.26 to 0.52)
Unknown	484/324	1.49 (1.37 to 1.63)	0.46 (0.34 to 0.59)
*P* value for heterogeneity		<.001	<.001
Adjusted (follow-up) *P* value for heterogeneity^b^		<.001	<.001

Abbreviations: AER, absolute excess risk; CNS, central nervous system; SMR, standardized mortality ratio.

^a^ Sex, follow-up period, age at death, deprivation, and ethnicity.

^b^ Fully adjusted for sex, cancer type, deprivation, race/ethnicity, age at cancer diagnosis, year of diagnosis, and follow-up period.

^c^ Fully adjusted for sex, cancer type, deprivation, ethnicity, age at cancer diagnosis, year of diagnosis, and attained age (age at death).

^d^ Estimate is based on a low number (<10) of observed events, and must be interpreted with caution.

### Variation in Suicide Risk by Last Primary Cancer

There was evidence of heterogeneity by cancer type for both SMRs and AERs (Table 2). This finding remained after adjustment for all factors of interest. The highest SMR and AER were observed among patients with mesothelioma, with a 4.51-fold risk compared with the general population, which corresponded to 4.20 extra deaths per 10 000 person-years. This risk was followed by pancreatic (SMR, 3.89; 95% CI, 2.77-5.48), esophageal (SMR, 2.65; 95% CI, 2.04-3.43), lung (SMR, 2.57; 95% CI, 2.23-2.97), and stomach (SMR, 2.20; 95% CI, 1.71-2.84) cancers. These excess risks resulted in between 1.35 and 2.89 extra deaths per 10 000 person-years for each cancer type. Patients with 6 further cancer types (including other malignant neoplasms) experienced a significantly increased risk of suicide in both absolute and relative terms. In contrast, patients with melanoma and prostate cancer had a significantly reduced risk of death by suicide compared with the general population.

### Variation in Suicide Risk by Sex

There were more than double the number of men who died by suicide compared with women (Table 1). The association with sex varied by cancer type and was greater when expressed in AERs (Table 4, eTable 4 in the Supplement). The AER for men was significantly greater than for women for 6 cancer types, namely, pancreas (men, 4.41; women, 1.37), esophagus (men, 2.40; women, 0.72), lung (men, 2.15; women, 0.86), stomach (men, 2.11; women, −0.05), head and neck (men, 1.08; women, 0.29), and colorectal (men, 0.47; women, 0.05). Significant heterogeneity (*P* = .03) was observed in the SMRs by sex among patients with stomach cancer (men, 2.46; women, 0.91).

**Table 4.** Suicide SMRs and AERs per 10 000 Person-Years at Risk According to Last Primary Cancer and Sex for the Primary Cancer Groupings With a Significantly Elevated SMR, Excluding Other Malignant Neoplasms^a^

Last Primary Cancer	Sex					
	SMR (95% CI)			AER per 10 000 (95% CI)		
	Men	Women	*P* Value for Heterogeneity	Men	Women	*P* Value for Heterogeneity
Mesothelioma	3.95 (2.42 to 6.45)	10.53 (3.95 to 28.05)^b^	.11	4.04 (1.39 to 6.70)	4.82 (−0.40 to 10.04)^b^	.79
Pancreas	3.99 (2.70 to 5.90)	3.62 (1.81 to 7.23)^b^	.81	4.41 (2.10 to 6.72)	1.37 (0.06 to 2.68)^b^	.02
Esophagus	2.69 (2.03 to 3.56)	2.41 (1.21 to 4.82)^b^	.77	2.40 (1.33 to 3.47)	0.72 (−0.13 to 1.57)^b^	.02
Lung	2.53 (2.14 to 2.99)	2.72 (2.03 to 3.64)	.68	2.15 (1.56 to 2.75)	0.86 (0.46 to 1.26)	<.001
Stomach	2.46 (1.89 to 3.20)	0.91 (0.34 to 2.42)^b^	.03	2.11 (1.17 to 3.05)	−0.05 (−0.52 to 0.42)^b^	<.001
Cancer of unknown primary	1.79 (1.14 to 2.81)	2.47 (1.33 to 4.59)	.42	1.23 (−0.02 to 2.48)	0.79 (−0.03 to 1.61)	.56
Head and neck	1.70 (1.45 to 2.01)	1.54 (1.10 to 2.15)	.58	1.08 (0.65 to 1.50)	0.29 (0.01 to 0.56)	.002
CNS (including brain)	1.50 (0.98 to 2.30)	2.04 (0.97 to 4.29)^b^	.49	0.88 (−0.25 to 2.01)	0.57 (−0.26 to 1.40)^b^	.67
Multiple myeloma	1.42 (0.99 to 2.05)	2.08 (1.18 to 3.67)	.28	0.61 (−0.14 to 1.36)	0.55 (−0.05 to 1.15)	.90
Colorectal	1.33 (1.18 to 1.50)	1.09 (0.86 to 1.38)	.13	0.47 (0.25 to 0.70)	0.05 (−0.09 to 0.18)	.001

Abbreviations: AER, absolute excess risk; CNS, central nervous system; SMR, standardized mortality ratio.

^a^ Observed and expected values are available in eTable 4 in the Supplement.

^b^ Estimate is based on a low number (<10) of observed events and must be interpreted with caution.

### Variation in Suicide Risk by Years Since Cancer Diagnosis

The SMR was greatest in the first 6 months after cancer diagnosis (SMR, 2.74; 95% CI, 2.52-2.98; AER, 1.77; 95% CI, 1.54-2.01), but remained elevated throughout the first 3 years (2-year SMR, 1.14; 95% CI, 1.01-1.28; AER, 0.13; 95% CI, 0.00-0.27). The risk decreased over time (*P* < .001). This association remained after multivariable adjustment (Figure, eTable 5 in the Supplement). The association with years since cancer diagnosis varied by cancer type, although there was a significantly increased risk in the first 6 months for all cancer types with an overall increased risk of suicide (eTable 4 and eTable 6 in the Supplement). Patients with mesothelioma had the highest risk of suicide in the first 6 months after diagnosis, with an 8.61-fold risk compared with the general population, corresponding to 9.36 extra deaths per 10 000 person-years at risk.

**Figure.**
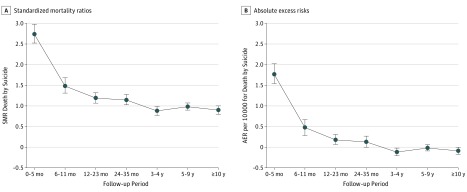
Suicide Standardized Mortality Ratios (SMRs) and Absolute Excess Risks (AERs) per 10 000 Person-Years at Risk by Follow-up Period Estimates of SMRs (A) and AERs (B) are presented in eTable 5 in the Supplement. Error bars indicate 95% CIs.

### Cumulative Risk of Suicide Mortality by Attained Age

The cumulative risk of suicide mortality was greatest for patients with mesothelioma, stomach, and lung cancer. By age 80 years, the cumulative risk was 1.60% for patients with mesothelioma. The corresponding expected cumulative mortality by this age was 0.63% (eFigure in the Supplement).

### Subgroup Analyses of Suicide Risk

Among patients with cancer diagnosed since 2012, significant heterogeneity was found by stage at diagnosis (*P* < .001) (eTable 7 in the Supplement). The SMR and AER were greatest for those with stage IV disease (SMR, 2.79; 95% CI, 2.24-3.47; AER, 1.97; 95% CI, 1.30-2.65).

## Discussion

In our nationwide population-based study of 4 722 099 patients, we found that being diagnosed with cancer in England confers a 20% increased risk of suicide compared with the general population, equivalent to 0.08% of all deaths among patients with cancer or 2491 potentially preventable deaths over this period. Our study identified specific factors characterizing those at higher risk, which will assist in the needs-based psychological assessment of these groups. Cancer types associated with particularly elevated risks are mesothelioma, pancreatic, lung, esophageal, and stomach cancers. These tend to be characterized by poor prognosis. We identified the first 6 months after a cancer diagnosis to be the period of highest risk. Although suicide is hard to predict, these findings indicate that all patients, and particularly those with the cancer types at highest risk, require improved psychological support and screening for suicidality in the immediate aftermath of cancer diagnosis.

In our cohort, we found only borderline differences by level of deprivation. Although deprivation is commonly understood to be an area-level risk factor for suicide, this is not a consistent association, and the divergent findings are likely to be explained by the size of the area of aggregation.^14^

### Findings in the Context of Other Studies

A number of studies have found an elevated risk of suicide in patients with cancer, in settings such as England, Finland, Sweden, Italy, Estonia, Japan, Norway, and the United States.^15^ Our findings are consistent with a previous analysis of local cancer registry data in England^16^ and European studies reported comparable SMRs, with similar high-risk clinical and demographic groups identified.^17,18^ However, an analysis of English primary care data found no elevated risk of suicide in patients with cancers overall or in men, but a 3-fold elevated risk of suicide in women, which was independent of clinical depression.^19^ The explanation for this apparent inconsistency in English findings may be that their data were derived from patients in 224 general physician practices, rather than our population coverage, with the validity of diagnoses reliant on quality and completeness of primary care physician records.^20^

The findings of cancer-specific analyses concur with ours in that patients with poor prognosis tend to be those at highest risk.^16^ More generally, the evidence suggests that suicide risk is associated with cancers with low survival rates^17^ and limited treatment options.^15^

We found that cancer risk varies by time since diagnosis, and the time bands that we chose suggested that suicide risk is highest in the first 6 months after diagnosis. Other studies have investigated differing time periods, but all found an elevated risk in the initial period defined after diagnosis, whether the first week,^21^ first month,^22^ or first year.^16^

### Strengths and Limitations

To our knowledge, this study is the first to investigate suicide risk in patients with cancer with entire population-based coverage in England. This strength should support responsive service provision in England. The advantages of complete cause of death ascertainment, national coverage, and precise diagnostic information reduced the biases introduced by missing data. Nevertheless, we acknowledge the limitation of the potential for underreporting of suicide as a cause of death.^23^ This lower level of reporting may affect data on patients with cancer more than the general population, with this bias therefore potentially underestimating the overall risk of suicide among patients with cancer.

Long-term follow-up over 22 million person-years at risk provided robust estimates of how risk varies by time since diagnosis. Tight definition of time periods clarified that risk concentrates within the first 6 months, providing more fine-grained risk information than the previous analysis of local English cancer registry data.^16^

The inclusion of race/ethnicity in cancer registry data allowed us to present the variation in suicide risk by race/ethnicity, unlike national suicide reports.^12^ Because the method of standardization could not include race/ethnicity, the observed trends are likely to reflect those in the general population. Given the extent of our population coverage, our findings provide the most representative description of race/ethnicity variation in suicide risk nationally, albeit with 32% missing data. Standardization was also not possible by deprivation quintile.

One limitation of the study is that it was not possible, due to current data availability, to adjust for preexisting psychiatric disorders or other potential confounders, such as alcohol or drug misuse diagnostic information. However, it has been demonstrated in a study of national cancer registration data that suicide risk is not explained by preexisting psychiatric conditions.^21^

An additional limitation is that, because of the small numbers of deaths, we were unable to perform multivariable regression models for specific cancer types or for the subgroup analysis to assess whether the trends observed could be explained by distributional differences in other variables measured. However, our multivariable model for all cancers combined suggested that variation in the patient characteristics were not explained by differences in the other variables, including cancer type.

### Clinical and Policy Implications

Clinically, our results identify specific cancers associated with significantly elevated risk of suicide and the first 6 months after diagnosis as being a critical period requiring greater vigilance for psychological distress and potential suicidality. The combined literature suggests that the first week after diagnosis has the highest risk for suicide, falling thereafter. This early phase is clearly a critical period. The mechanisms of suicide among patients with cancer are likely to be heterogeneous, involving factors beyond diagnosable psychiatric disorder. The shock of a cancer diagnosis and the losses inherent to it may be processed as a psychological threat. In some cases this shock can give rise to psychological symptoms diagnosable as depression or anxiety. The prevalence of major depression in patients with cancers is 15%,^24^ exceeding the 2% reported for the general population.^25^ However, as diagnostic criteria for depression rely on at least 2 weeks of pervasive low mood, patients who attempt suicide within a week of a cancer diagnosis may have been depressed for some time^26^ or enacting a catastrophic reaction to a negative life event.^27^

Specific cancers may present insidiously with depressed mood and certain treatments for cancer can also cause depression through their direct neuropsychiatric effects.^1^ Awareness of the potential for such biological effects, impulsive catastrophic reactions to diagnosis, and incident or worsening depression are starting points for early identification of suicidal distress.

Predicting suicide is difficult, and most people identified as high risk for suicide do not die by suicide.^28^ English patients with cancer have a low ratio of self-harm events to suicides compared with those for other physical and psychiatric disorders^29^ and the general population,^30^ presenting fewer early warning signs for suicide and limiting opportunities for intervention. Nevertheless, our findings identify the timeframe over which health professionals should ensure better monitoring of the psychological health of all patients with cancer, and particularly those with poor prognoses, and address modifiable risk factors, such as pain, substance use disorders, and psychiatric illness. Efforts to address the underdiagnosis and undertreatment of depression and anxiety in patients with cancer are likely to reap benefits in terms of reducing suicide risk, as well as positively influencing quality of life, treatment adherence, and treatment costs.^1^ Models that integrate psychological support into cancer care, specifically collaborative screening and treatment, have shown benefits in terms of reduced depression, anxiety, and pain, even if they lack evidence for survival benefits.^31^

Access to the means of suicide may influence risk, and restricting access is the suicide prevention intervention with the best evidence for effectiveness.^32^ This suggests that, for patients with cancer who are thought to be at higher risk of suicide, there may be scope to consider effective pain management options other than opioids,^33^ transdermal administration routes, or to involve caregivers in medication administration and safeguarding.

### Future Research

Our study used a lower age limit of 18 years, and the numbers of suicides in patients with cancers younger than 18 years were low. We were therefore unable to investigate suicide risk in teenagers and young adults, among whom evidence is limited. Pooling data could help to address this gap, as could using statistical methods, such as the self-controlled case series approach, to identify the precise points at which suicide risk is elevated.^34^ Further research could also consider hospital admissions for suicide attempts or the presence of preexisting or intercurrent psychiatric illness to support the identification of modifiable risk factors.

## Conclusions

We found an elevated risk of suicide in patients with cancer, particularly in the first 6 months after diagnosis. Patients with mesothelioma, pancreatic, lung, esophageal, and stomach cancers had the highest risk. The increased risk of suicide persisted for 3 years after diagnosis, identifying a period during which cancer care pathways should pay attention to the psychological health needs of the patients. Suggested responses include addressing the underdiagnosis and undertreatment of depression and anxiety in patients with cancer, improving access to integrated psychological support for all patients with cancers, addressing modifiable risk factors, and restricting access to the means of lethal overdose.

## Notes

Supplement. **eTable 1.** Cancer Type Classification Using International Statistical Classification of Diseases and Related Health Problems 10th Revision **eTable 2.** Relative Risks and Excess Mortality Ratios According to Key Patient Characteristics for All Cancers Combined, Namely Sex, Last Primary Cancer, Follow-up Period, Age at Death, Deprivation, Age at Cancer Diagnosis, Year of Cancer Diagnosis and Ethnicity From a Multivariable Poisson Regression Model Adjusted for the Specified Potential Confounders **eTable 3.** Suicide Standardised Mortality Ratios and Absolute Excess Risks per 10 000 Person-Years at Risk According to Both Last Primary Cancer and Age at Death for the Primary Cancer Groupings With a Significantly Elevated SMR (Excluding Other Malignant Neoplasms) **eTable 4.** Observed and Expected Number of Suicides by Last Primary Cancer, Sex and Follow-up Period for the Primary Cancer Groupings With a Significantly Elevated Standardised Mortality Ratio (Excluding Other Malignant Neoplasms) **eTable 5.** Suicide Standardised Mortality Ratios and Absolute Excess Risks per 10 000 Person-Years at Risk According to Follow-up Period for All Cancers Combined **eTable 6.** Suicide Standardised Mortality Ratios and Absolute Excess Risks per 10 000 Person-Years at Risk According to Both Last Primary Cancer and Years Since Cancer Diagnosis for the Primary Cancer Groupings With a Significantly Elevated SMR **eTable 7.** Subgroup Analysis of Suicide Standardised Mortality Ratios and Absolute Excess Risks per 10 000 Person-Years at Risk According to Stage of Cancer Diagnosis for Those Patients Diagnosed Since 2012 **eFigure.** Cumulative Mortality Due to Suicide Among Patients With Cancer Between 1995 and 2015 in England According to Attained Age for the Primary Cancer Groupings With a Significantly Elevated Standardised Mortality Ratio and Absolute Excess Risk
