# Supplementary material for: Risk of Suicide After Cancer Diagnosis in England
Source: JAMA Psychiatry. 2018 Nov 21;76(1):51–60. doi: 10.1001/jamapsychiatry.2018.3181 (PMC6583458; doi:10.1001/jamapsychiatry.2018.3181)
Supplement: Supplement. — eTable 1. Cancer Type Classification Using International Statistical Classification of Diseases and Related Health Problems 10th Revision eTable 2. Relative Risks and Excess Mortality Ratios According to Key Patient Characteristics for All Cancers Combined, Namely Sex, Last Primary Cancer, Follow-up Period, Age at Death, Deprivation, Age at Cancer Diagnosis, Year of Cancer Diagnosis and Ethnicity From a Multivariable Poisson Regression Model Adjusted for the Specified Potential Confounders eTable 3. Suicide Standardised Mortality Ratios and Absolute Excess Risks per 10 000 Person-Years at Risk According to Both Last Primary Cancer and Age at Death for the Primary Cancer Groupings With a Significantly Elevated SMR (Excluding Other Malignant Neoplasms) eTable 4. Observed and Expected Number of Suicides by Last Primary Cancer, Sex and Follow-up Period for the Primary Cancer Groupings With a Significantly Elevated Standardised Mortality Ratio (Excluding Other Malignant Neoplasms) eTable 5. Suicide Standardised Mortality Ratios and Absolute Excess Risks per 10 000 Person-Years at Risk According to Follow-up Period for All Cancers Combined eTable 6. Suicide Standardised Mortality Ratios and Absolute Excess Risks per 10 000 Person-Years at Risk According to Both Last Primary Cancer and Years Since Cancer Diagnosis for the Primary Cancer Groupings With a Significantly Elevated SMR eTable 7. Subgroup Analysis of Suicide Standardised Mortality Ratios and Absolute Excess Risks per 10 000 Person-Years at Risk According to Stage of Cancer Diagnosis for Those Patients Diagnosed Since 2012 eFigure. Cumulative Mortality Due to Suicide Among Patients With Cancer Between 1995 and 2015 in England According to Attained Age for the Primary Cancer Groupings With a Significantly Elevated Standardised Mortality Ratio and Absolute Excess Risk [file jamapsychiatry-76-51-s001.pdf]

Supplementary Online Content

Henson KE, Brock R, Charnock J, Wickramasinghe B, Will O, Pitman A. Risk of suicide after cancer diagnosis in England. *JAMA Psychiatry*. Published online November 21, 2018. doi:10.1001/jamapsychiatry.2018.3181

eTable 1. Cancer Type Classification Using International Statistical Classification of Diseases and Related Health Problems 10th Revision

eTable 2. Relative Risks and Relative Excess Risks According to Key Patient Characteristics for All Cancers Combined, Namely Sex, Last Primary Cancer, Follow-up Period, Age at Death, Deprivation, Age at Cancer Diagnosis, Year of Cancer Diagnosis and Ethnicity From a Multivariable Poisson Regression Model Adjusted for the Specified Potential Confounders

eTable 3. Suicide Standardised Mortality Ratios (SMR) and Absolute Excess Risks per 10,000 Person-Years at Risk According to Both Last Primary Cancer and Attained Age for the Primary Cancer Groupings With a Significantly Elevated SMR (Excluding Other Malignant Neoplasms)

eTable 4. Observed and Expected Number of Suicides by Last Primary Cancer, Sex and Follow-up Period for the Primary Cancer Groupings With a Significantly Elevated Standardised Mortality Ratio (Excluding Other Malignant Neoplasms)

eTable 5. Suicide Standardised Mortality Ratios and Absolute Excess Risks per 10,000 Person-Years at Risk According to Follow-up Period for All Cancers Combined

eTable 6. Suicide Standardised Mortality Ratios (SMR) and Absolute Excess Risks per 10,000 Person-Years at Risk According to Both Last Primary Cancer and Years Since Cancer Diagnosis for the Primary Cancer Groupings With a Significantly Elevated SMR

eTable 7. Subgroup Analysis of Suicide Standardised Mortality Ratios and Absolute Excess Risks per 10,000 Person-Years at Risk According to Stage of Cancer Diagnosis for Those Patients Diagnosed Since 2012

eFigure. Cumulative Mortality Due to Suicide Among Patients With Cancer Between 1995 and 2015 in England According to Attained Age for the Primary Cancer Groupings With a Significantly Elevated Standardised Mortality Ratio and Absolute Excess Risk

This supplementary material has been provided by the authors to give readers additional information about their work.

© 2018 Henson KE et al. *JAMA Psychiatry.*

eTable 1: Cancer Type Classification Using International Statistical Classification of Diseases and Related Health Problems 10th Revision (ICD-10).

**Cancer Type**	**ICD-10**
Bladder	C67
Breast	C50
Cancer of Unknown Primary	C77 – C80
Central Nervous System (incl brain)	C70- C72
Cervix	C53
Colorectal	C18 – C20
Head and neck	C00 – C14, C31 – C32, C73
Hodgkin lymphoma	C81
Kidney and unspecified urinary organs	C64 – C66, C68
Leukaemia	C91 – C95
Liver	C22
Lung	C33 – C34
Melanoma	C43
Mesothelioma	C45
Multiple myeloma	C88, C90
Non-Hodgkin lymphoma	C82 – C85
Oesophagus	C15
Other malignant neoplasms	C17, C21, C23, C24, C26, C30, C37, C38, C39, C46, C47, C48, C51, C52, C58, C60, C63, C69, C74, C75, C76, C96, C97
Ovary	C56 – C57
Pancreas	C25
Prostate	C61
Sarcoma	C40 – C41, C49
Stomach	C16
Testis	C62
Uterus	C54 – C55

© 2018 Henson KE et al. *JAMA Psychiatry.*

eTable 2. Relative Risks (RR) and Relative Excess Risks (RER) According to Key Patient Characteristics for All Cancers Combined, Namely Sex, Last Primary Cancer, Follow-up Period, Age at Death, Deprivation, Age at Cancer Diagnosis, Year of Cancer Diagnosis and Ethnicity From a Multivariable Poisson Regression Model Adjusted for the Specified Potential Confounders.

	**Follow-up**		**Attained age**	
	**RR [a] (95% CI[b])**	**RER [c] (95% CI)**	**RR [a] (95% CI[b])**	**RER [c] (95% CI)**
**Last Primary Cancer**				
Bladder	0.96 (0.78 - 1.18)	1.09 (0.64 - 1.88)	0.96 (0.78 - 1.18)	1.10 (0.58 - 2.05)
Breast	0.90 (0.75 - 1.07)	**0.50 (0.26 - 0.96)**	0.88 (0.74 - 1.05)	**0.45 (0.22 - 0.92)**
Cancer of Unknown Primary	1.34 (0.92 - 1.96)	**2.41 (1.36 - 4.26)**	**1.54 (1.05 - 2.25)**	**3.64 (1.95 - 6.81)**
Central Nervous System (incl brain)	1.33 (0.90 - 1.97)	1.55 (0.69 - 3.48)	**1.48 (1.00 - 2.19)**	**2.64 (1.07 - 6.53)**
Cervix	0.99 (0.64 - 1.53)	1.62 (0.50 - 5.19)	0.97 (0.62 - 1.50)	1.60 (0.43 - 5.90)
Colorectal	*ref*	*ref*	*ref*	*ref*
Head and neck	**1.39 (1.15 - 1.67)**	**2.18 (1.42 - 3.34)**	**1.38 (1.15 - 1.66)**	**2.38 (1.50 - 3.78)**
Hodgkin lymphoma	0.99 (0.65 - 1.50)	[f]	0.96 (0.63 - 1.46)	[f]
Kidney and unspecified urinary organs	0.96 (0.74 - 1.24)	1.14 (0.63 - 2.06)	0.96 (0.75 - 1.24)	1.03 (0.50 - 2.13)
Leukaemia	0.82 (0.62 - 1.10)	0.66 (0.27 - 1.60)	0.83 (0.62 - 1.11)	0.90 (0.39 - 2.09)
Liver	0.99 (0.53 - 1.86)	0.61 (0.11 - 3.35)	1.15 (0.61 - 2.16)	1.17 (0.22 - 6.16)
Lung	**1.57 (1.31 - 1.88)**	**2.25 (1.58 - 3.21)**	**1.85 (1.55 - 2.21)**	**3.76 (2.53 - 5.59)**
Melanoma	**0.66 (0.53 - 0.83)**	[f]	**0.64 (0.51 - 0.81)**	[f]
Mesothelioma	**2.44 (1.55 - 3.84)**	**3.13 (1.67 - 5.87)**	**3.09 (1.96 - 4.85)**	**6.28 (3.26 - 12.08)**
Multiple myeloma	1.20 (0.87 - 1.66)	1.10 (0.49 - 2.48)	1.22 (0.89 - 1.69)	1.06 (0.38 - 2.96)
Non-Hodgkin lymphoma	1.02 (0.83 - 1.27)	0.98 (0.53 - 1.80)	1.01 (0.82 - 1.25)	1.19 (0.64 - 2.21)
Oesophagus	**1.68 (1.27 - 2.23)**	**2.08 (1.25 - 3.47)**	**1.94 (1.47 - 2.57)**	**3.77 (2.24 - 6.35)**
Other malignant neoplasms	1.18 (0.92 - 1.51)	1.25 (0.66 - 2.37)	1.19 (0.93 - 1.53)	1.56 (0.82 – 3.00)
Ovary	0.98 (0.68 - 1.42)	0.43 (0.03 - 5.40)	0.99 (0.69 - 1.44)	0.91 (0.25 - 3.27)
Pancreas	**2.20 (1.53 - 3.15)**	**2.69 (1.54 - 4.70)**	**2.80 (1.96 - 4.00)**	**5.94 (3.35 - 10.52)**
Prostate	**0.67 (0.58 - 0.78)**	**0.24 (0.13 - 0.45)**	**0.66 (0.57 - 0.76)**	**0.23 (0.11 - 0.48)**
Sarcoma	0.68 (0.40 - 1.17)	[f]	0.68 (0.40 - 1.17)	[f]
Stomach	**1.55 (1.17 - 2.04)**	**2.32 (1.40 - 3.84)**	**1.72 (1.30 - 2.26)**	**3.33 (1.91 - 5.79)**
Testis	0.85 (0.63 - 1.15)	[f]	0.81 (0.60 - 1.10)	[f]
Uterus	0.71 (0.50 - 1.02)	0.57 (0.16 - 1.99)	0.70 (0.49 - 1.00)	0.17 (0.00 – 9.22)
Adjusted 2p for heterogeneity [d, e]	***<0.0001***	***<0.0001***	***<0.0001***	***<0.0001***
**Sex**				
Male	*ref*	*ref*	*ref*	*ref*
Female	1.04 (0.92 - 1.17)	**0.35 (0.27 - 0.46)**	1.04 (0.92 - 1.17)	**0.37 (0.28 - 0.48)**
Adjusted 2p for heterogeneity [d, e]	*0.52*	***<0.0001***	*0.57*	***<0.0001***
**By follow-up period**				
0-5 months	**0.58 (0.50 - 0.67)**	**0.39 (0.29 - 0.52)**		
6-11 months	**0.49 (0.42 - 0.56)**	**0.20 (0.13 - 0.30)**		
12-23 months	**0.48 (0.41 - 0.56)**	**0.18 (0.11 - 0.29)**		
24-35 months	**0.38 (0.33 - 0.43)**	**0.03 (0.00 - 0.17)**		
3-4 years	**0.43 (0.38 - 0.49)**	**0.03 (0.01 - 0.12)**		
5-9 years	*ref*	*ref*		
10+ years	**0.45 (0.38 - 0.52)**	[f]		
Adjusted 2p for heterogeneity [d]	***<0.0001***	***<0.0001***		

© 2018 Henson KE et al. *JAMA Psychiatry.*

**Age at death (attained age)**				
18-29 yrs			1.04 (0.54 – 2.03)	
30-49 yrs			**1.36 (1.04 - 1.78)**	[f]
50-59 yrs			**1.22 (1.03 - 1.46)**	[f]
60-69 yrs			*ref*	*ref*
70-79 yrs			**0.79 (0.67 - 0.92)**	[f]
80+ yrs			**0.56 (0.45 - 0.70)**	[f]
Adjusted 2p for heterogeneity[e]			***<0.0001***	[f]
**Deprivation**				
1 – least deprived	*ref*	*ref*	*ref*	*ref*
2	1.08 (0.96 - 1.22)	0.91 (0.66 - 1.25)	1.09 (0.96 - 1.23)	0.99 (0.71 - 1.38)
3	1.08 (0.95 - 1.22)	0.87 (0.62 - 1.20)	1.08 (0.96 - 1.23)	0.87 (0.61 - 1.24)
4	**1.14 (1.00 - 1.29)**	1.09 (0.80 - 1.47)	**1.14 (1.01 - 1.30)**	1.15 (0.83 - 1.59)
5 – most deprived	1.12 (0.98 - 1.28)	0.82 (0.58 - 1.15)	1.13 (0.99 - 1.29)	0.96 (0.67 - 1.38)
Adjusted 2p for heterogeneity [d, e]	*0.32*	*0.43*	*0.25*	*0.61*
**Ethnicity**				
White	*ref*	*ref*	*ref*	*ref*
Mixed	1.22 (0.51 - 2.93)	2.79 (0.71 - 11.04)	1.22 (0.51 - 2.94)	2.92 (0.67 - 12.60)
Asian	**0.53 (0.33 - 0.87)**	[f]	**0.53 (0.32 - 0.87)**	[f]
Black	**0.42 (0.23 - 0.76)**	[f]	**0.42 (0.23 - 0.76)**	[f]
Other	1.06 (0.67 - 1.66)	1.57 (0.54 - 4.58)	1.05 (0.67 - 1.66)	1.65 (0.56 - 4.86)
Not Stated	**1.53 (1.37 - 1.71)**	**2.51 (1.87 - 3.37)**	**1.55 (1.39 - 1.74)**	**2.82 (2.09 - 3.80)**
Unknown	**1.50 (1.35 - 1.66)**	**2.82 (2.22 - 3.58)**	**1.51 (1.36 - 1.67)**	**2.95 (2.29 - 3.79)**
Adjusted 2p for heterogeneity [d, e]	*<0.0001*	*<0.0001*	*<0.0001*	*<0.0001*
**Age at cancer diagnosis**				
18-29 yrs	**0.67 (0.49 - 0.92)**	[f]	**0.46 (0.29 - 0.72)**	[f]
30-49 yrs	**0.75 (0.65 - 0.86)**	0.79 (0.49 - 1.28)	**0.50 (0.38 - 0.64)**	**0.01 (0.00 - 0.24)**
50-59 yrs	0.93 (0.83 - 1.05)	0.94 (0.68 - 1.31)	**0.74 (0.63 - 0.88)**	**0.10 (0.01 - 0.62)**
60-69 yrs	*ref*	*ref*	*ref*	*ref*
70-79 yrs	1.02 (0.91 - 1.13)	0.96 (0.74 - 1.25)	**1.33 (1.14 - 1.55)**	[f]
80+ yrs	0.94 (0.82 - 1.07)	1.04 (0.76 - 1.40)	**1.65 (1.32 - 2.06)**	[f]
Adjusted 2p for heterogeneity [d, e]	***0.0002***	***0.06***	***<0.0001***	***<0.0001***
**Decade of cancer diagnosis**				
1995-1999	**0.61 (0.54 - 0.69)**	[f]	**0.62 (0.55 - 0.69)**	[f]
2000-2004	*ref*	*ref*	*ref*	*ref*
2005-2009	1.05 (0.94 - 1.17)	1.19 (0.91 - 1.55)	1.02 (0.92 - 1.14)	1.14 (0.87 - 1.49)
2010-2015	1.03 (0.92 - 1.16)	1.13 (0.86 - 1.48)	1.06 (0.95 - 1.19)	1.06 (0.80 - 1.41)
Adjusted 2p for heterogeneity [d, e]	***<0.0001***	***<0.0001***	***<0.0001***	***<0.0001***

[a] relative risks – can be interpreted as ratios of standardised mortality ratios adjusted for confounding risk factors included in the model

[b] confidence interval

[c] relative excess risks - can be interpreted as ratios of absolute excess risks adjusted for confounding risk factors included in the model

[d] fully adjusted for sex, cancer type, deprivation, ethnicity, age at cancer diagnosis, year of diagnosis, and follow-up period

[e] fully adjusted for sex, cancer type, deprivation, ethnicity, age at cancer diagnosis, year of diagnosis, and attained age (age at death)

[f] unreliable model fit due to small numbers of events

Statistically significant estimates are presented in bold.

© 2018 Henson KE et al. *JAMA Psychiatry.*

© 2018 Henson KE et al. *JAMA Psychiatry.*

eTable 3. Suicide Standardised Mortality Ratios (SMRs) and Absolute Excess Risks (AERs) per 10,000 Person-Years at Risk According to Both Last Primary Cancer and Attained Age for the Primary Cancer Groupings With a Significantly Elevated SMR (excluding other malignant neoplasms).

**Last Primary Cancer**	**Age at death (attained age)**										
	**Observed / Expected**			**SMR [a] (95% CI[b])**			***p* [d]**	**AER [c] per 10,000 (95% CI)**			***p* [d]**
	**18-59 yrs**	**60-69 yrs**	**70+ yrs**	**18-59 yrs**	**60-69 yrs**	**70+ yrs**		**18-59 yrs**	**60-69 yrs**	**70+ yrs**	
Mesothelioma	3 / 1	6 / 1	11 / 2	**3.37 ***	**4.62 ***	**4.91**	*0.83*	3.54 *	3.95 *	**4.57**	*0.83*
				**(1.09 - 10.44)**	**(2.07 - 10.27)**	**(2.72 - 8.87)**		(-2.16 - 9.23)	(-0.08 - 7.98)	**(1.18 - 7.95)**	
Pancreas	9 / 3	8 / 2	16 / 4	**3.32 ***	**3.73 ***	**4.42**	*0.78*	**3.05 ***	**2.46 ***	**3.07**	*0.78*
				**(1.73 - 6.38)**	**(1.86 - 7.46)**	**(2.71 - 7.21)**		**(0.20 - 5.89)**	**(0.13 - 4.78)**	**(1.12 - 5.01)**	
Oesophagus	8 / 5	16 / 6	33 / 10	1.48 *	**2.69**	**3.25**	*0.10*	0.74 *	**1.74**	**2.27**	*0.10*
				(0.74 - 2.96)	**(1.65 - 4.40)**	**(2.31 - 4.56)**		(-0.84 - 2.33)	**(0.38 - 3.09)**	**(1.15 - 3.39)**	
Lung	38 / 15	55 / 19	91 / 38	**2.62**	**2.96**	**2.37**	*0.44*	**2.04**	**1.75**	**1.33**	*0.44*
				**(1.91 - 3.60)**	**(2.27 - 3.85)**	**(1.93 - 2.91)**		**(0.99 - 3.09)**	**(1.05 - 2.45)**	**(0.86 - 1.80)**	
Stomach	14 / 6	15 / 6	30 / 16	**2.54**	**2.71**	**1.91**	*0.46*	**2.25**	**1.73**	**0.98**	*0.46*
				**(1.51 - 4.29)**	**(1.63 - 4.50)**	**(1.33 - 2.73)**		**(0.31 - 4.19)**	**(0.34 - 3.12)**	**(0.24 - 1.71)**	
Cancer of Unknown Primary	10 / 5	4 / 3	15 / 6	1.82	1.29 *	**2.48**	*0.44*	1.07	0.25 *	**1.36**	*0.44*
				(0.98 - 3.38)	(0.48 - 3.43)	**(1.50 - 4.12)**		(-0.40 - 2.53)	(-0.85 - 1.36)	**(0.21 - 2.51)**	
Head and neck	69 / 49	48 / 25	59 / 31	**1.40**	**1.92**	**1.90**	*0.12*	**0.50**	**0.90**	**0.91**	*0.12*
				**(1.10 - 1.77)**	**(1.45 - 2.55)**	**(1.47 - 2.45)**		**(0.08 - 0.91)**	**(0.37 - 1.43)**	**(0.42 - 1.39)**	
Central Nervous System	20 / 14	3 / 2	5 / 1	1.46	1.30 *	**3.46 ***	*0.27*	0.63	0.28 *	2.16 *	*0.27*
				(0.94 - 2.27)	(0.42 - 4.04)	**(1.44 - 8.31)**		(-0.24 - 1.50)	(-1.09 - 1.65)	(-0.50 - 4.81)	
Multiple myeloma	14 / 7	10 / 7	17 / 13	**2.01**	1.52	1.35	*0.54*	1.42	0.48	0.33	*0.54*
				**(1.19 - 3.39)**	(0.82 - 2.83)	(0.84 - 2.16)		(-0.06 - 2.90)	(-0.38 - 1.34)	(-0.28 - 0.94)	
Colorectal	67 / 49	79 / 59	203 / 165	**1.35**	**1.33**	**1.23**	*0.72*	**0.47**	**0.31**	**0.22**	*0.72*
				**(1.07 - 1.72)**	**(1.07 - 1.66)**	**(1.07 - 1.41)**		**(0.04 - 0.89)**	**(0.04 - 0.58)**	**(0.06 - 0.39)**	

[a] standardised mortality ratio
[b] confidence interval
[c] absolute excess risk
[d] two-sided p for heterogeneity
* estimate is based on a low number (<10) of observed events, and must be interpreted with caution
Statistically significant estimates are presented in bold.

© 2018 Henson KE et al. *JAMA Psychiatry.*

eTable 4. Observed and Expected Number of Suicides by Last Primary Cancer, Sex and Follow-up Period for the Primary Cancer Groupings With a Significantly Elevated Standardised Mortality Ratio (SMR) (excluding other malignant neoplasms). *SMR and AER (Absolute Excess Risk) estimates are available in Table 4 and eTable 5.*

Last Primary Cancer	Sex		Years since cancer diagnosis		
	Male	Female	0-5 months	6-11 months	1+ years
Mesothelioma	16 / 4	4 / 0	14 / 2	3 / 1	3 / 2
Pancreas	25 / 6	8 / 2	18 / 3	6 / 1	9 / 4
Oesophagus	49 / 18	8 / 3	21 / 5	10 / 3	26 / 13
Lung	139 / 55	45 / 17	105 / 20	30 / 11	49 / 41
Stomach	55 / 22	4 / 4	27 / 5	7 / 3	25 / 18
Cancer of Unknown Primary	19 / 11	10 / 4	18 / 3	2 / 1	9 / 10
Head and neck	142 / 83	34 / 22	40 / 9	16 / 8	120 / 88
Central Nervous System (incl brain)	21 / 14	7 / 3	13 / 3	2 / 2	13 / 13
Multiple myeloma	29 / 20	12 / 6	11 / 3	5 / 3	25 / 20
Colorectal	280 / 210	69 / 63	73 / 26	28 / 22	248 / 226

© 2018 Henson KE et al. *JAMA Psychiatry.*

eTable 5. Suicide Standardised Mortality Ratios (SMRs) and Absolute Excess Risks (AERs) per 10,000 Person-Years at Risk According to Follow-up Period for All Cancers Combined. *SMR and AER estimates are presented in Figure 1.*

By follow-up period	Observed / Expected	SMR [a] (95% CI[b])	AER [c] per 10,000 (95% CI)
0-5 months	540 / 197	**2.74 (2.52 - 2.98)**	**1.77 (1.54 - 2.01)**
6-11 months	241 / 163	**1.48 (1.31 - 1.68)**	**0.48 (0.29 - 0.67)**
1- years	329 / 277	**1.19 (1.07 - 1.32)**	**0.18 (0.06 - 0.31)**
2- years	261 / 230	**1.14 (1.01 - 1.28)**	0.13 (-0.00 - 0.27)
3-4 years	316 / 360	**0.88 (0.79 - 0.98)**	**-0.12 (-0.21 - -0.02)**
5-9 years	521 / 531	0.98 (0.90 - 1.07)	-0.02 (-0.09 - 0.06)
10+ years	283 / 314	0.90 (0.80 - 1.01)	-0.09 (-0.17 - 0.00)
	2p for heterogeneity	***<0.0001***	***<0.0001***
	Adjusted (follow-up) 2p for heterogeneity [d]	***<0.0001***	***<0.0001***

[a] standardised mortality ratio
[b] confidence interval
[c] absolute excess risk
[d] fully adjusted for sex, cancer type, deprivation, ethnicity, age at cancer diagnosis, year of diagnosis, and follow-up period
Statistically significant estimates are presented in bold.

© 2018 Henson KE et al. *JAMA Psychiatry.*

eTable 6: Suicide Standardised Mortality Ratios (SMRs) and Absolute Excess Risks (AERs) per 10,000 Person-Years at Risk According to Both Last Primary Cancer and Years Since Cancer Diagnosis for the Primary Cancer Groupings With a Significantly Elevated SMR (excluding other malignant neoplasms). *Observed and expected values are available in eTable 4.*

	**Years since cancer diagnosis**							
	**SMR [a] (95% CI[b])**				**AER [c] per 10,000 (95% CI)**			
	**0-5 months**	**6-11 months**	**1+ years**	***2p for heterogeneity***	**0-5 months**	**6-11 months**	**1+ years**	***2p for heterogeneity***
Mesothelioma	**8.61**	**3.23 ***	1.60 *	***0.007***	**9.36**	2.68 *	0.70 *	***0.006***
	**(5.10 - 14.54)**	**(1.04 - 10.01)**	(0.52 - 4.96)		**(3.81 - 14.91)**	(-1.72 - 7.08)	(-1.41 - 2.80)	
Pancreas	**5.75**	**4.45 ***	**2.25 ***	*0.06*	**4.77**	3.45 *	1.24 *	***0.05***
	**(3.62 - 9.13)**	**(2.00 - 9.90)**	**(1.17 - 4.32)**		**(2.10 - 7.44)**	(-0.11 - 7.01)	(-0.22 - 2.71)	
Oesophagus	**3.97**	**3.14**	**1.99**	*0.06*	**3.46**	**2.48**	**1.08**	***0.04***
	**(2.59 - 6.09)**	**(1.69 - 5.84)**	**(1.36 - 2.93)**		**(1.48 - 5.43)**	**(0.23 - 4.73)**	**(0.25 - 1.91)**	
Lung	**5.28**	**2.79**	1.20	***<0.0001***	**4.51**	**1.83**	0.19	***<0.0001***
	**(4.36 - 6.40)**	**(1.95 - 3.99)**	(0.91 - 1.59)		**(3.45 - 5.58)**	**(0.81 - 2.85)**	(-0.13 - 0.51)	
Stomach	**5.18**	**2.18 ***	1.36	***<0.0001***	**4.94**	1.37 *	0.40	***<0.0001***
	**(3.55 - 7.55)**	**(1.04 - 4.57)**	(0.92 - 2.02)		**(2.63 - 7.25)**	(-0.51 - 3.25)	(-0.19 - 0.99)	
Cancer of Unknown Primary	**5.33**	1.36 *	0.92 *	***<0.0001***	**4.47**	0.37 *	-0.08 *	***<0.0001***
	**(3.36 - 8.46)**	(0.34 - 5.45)	(0.48 - 1.77)		**(1.93 - 7.02)**	(-1.57 - 2.32)	(-0.69 - 0.53)	
Head and neck	**4.40**	**1.98**	**1.36**	***<0.0001***	**4.01**	**1.15**	**0.39**	***<0.0001***
	**(3.23 - 5.99)**	**(1.22 - 3.24)**	**(1.14 - 1.63)**		**(2.40 - 5.62)**	**(0.01 - 2.29)**	**(0.13 - 0.65)**	
Central Nervous System (incl brain)	**4.45**	1.08 *	1.03	***0.001***	**4.06**	0.10 *	0.03	***0.002***
	**(2.58 - 7.67)**	(0.27 - 4.33)	(0.60 - 1.77)		**(1.21 - 6.91)**	(-1.75 - 1.96)	(-0.66 - 0.72)	
Multiple myeloma	**3.36**	1.79 *	1.24	***0.04***	**2.50**	0.83 *	0.25	***0.03***
	**(1.86 - 6.07)**	(0.75 - 4.31)	(0.84 - 1.84)		**(0.40 - 4.60)**	(-0.82 - 2.48)	(-0.25 - 0.75)	
Colorectal	**2.82**	1.26	1.10	***<0.0001***	**1.93**	0.28	0.10	***<0.0001***
	**(2.24 - 3.55)**	(0.87 - 1.83)	(0.97 - 1.25)		**(1.24 - 2.62)**	(-0.22 - 0.77)	(-0.04 - 0.24)	

[a] standardised mortality ratio
[b] confidence interval
[c] absolute excess risk
* estimate is based on a low number (<10) of observed events, and must be interpreted with caution
Statistically significant estimates are presented in bold.

© 2018 Henson KE et al. *JAMA Psychiatry.*

eTable 7. Subgroup Analysis of Suicide Standardised Mortality Ratios (SMRs) and Absolute Excess Risks (AERs) per 10,000 Person-Years at Risk According to Stage of Cancer Diagnosis for Those Patients Diagnosed Since 2012.

	Observed / Expected	SMR [a] (95% CI[b])	AER [c] per 10,000 (95% CI)
**Total**	347 / 239	**1.45**	**0.43**
		**(1.30 - 1.61)**	**(0.29 - 0.58)**
**Stage at cancer diagnosis**			
1	68 / 72	0.95	-0.05
		(0.75 - 1.20)	(-0.25 - 0.15)
2	57 / 44	1.29	0.25
		(0.99 - 1.67)	(-0.04 - 0.55)
3	48 / 36	**1.35**	0.35
		**(1.02 - 1.79)**	(-0.03 - 0.74)
4	80 / 29	**2.79**	**1.97**
		**(2.24 - 3.47)**	**(1.30 - 2.65)**
Unknown	94 / 59	**1.59**	**0.62**
		**(1.30 - 1.95)**	**(0.28 - 0.95)**
	2p for heterogeneity	***<0.0001***	***<0.0001***

[a] standardised mortality ratio
[b] confidence interval
[c] absolute excess risk
Statistically significant estimates are presented in bold.

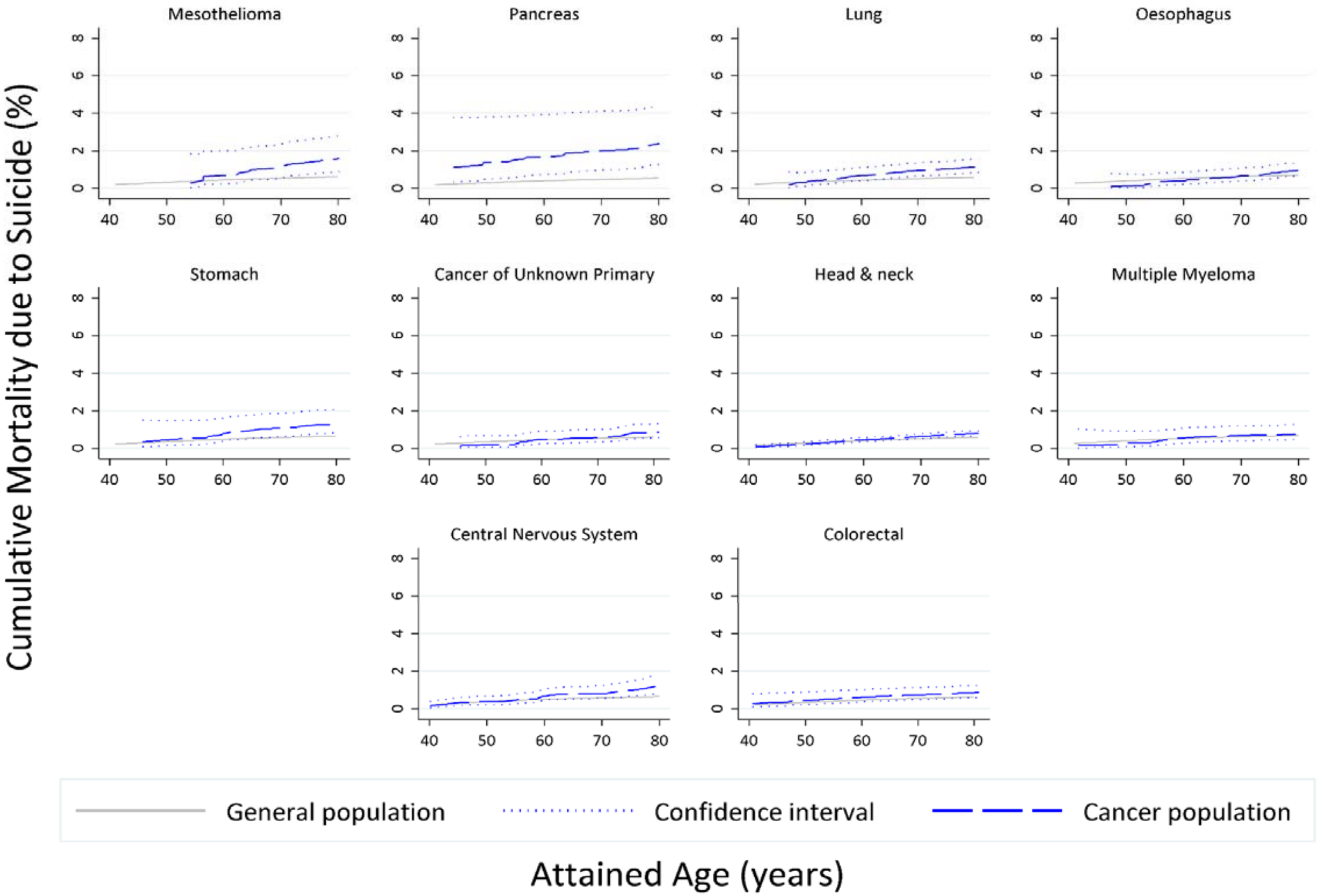
Mesothelioma
Pancreas
Lung
Oesophagus
Stomach
Cancer of Unknown Primary
Head & neck
Multiple Myeloma
Central Nervous System
Colorectal
Cumulative Mortality due to Suicide (%)
General population
Confidence interval
Cancer population
Attained Age (years)

eFigure. Cumulative Mortality Due to Suicide Among Patients With Cancer Between 1995 and 2015 in England According to Attained Age for the Primary Cancer Groupings With a Significantly Elevated Standardised Mortality Ratio (SMR) and Absolute Excess Risk (AER) (excluding other malignant neoplasms).
